# Predictors of smoking relapse in a cohort of adolescents and young adults in Monastir (Tunisia)

**DOI:** 10.1186/1617-9625-11-12

**Published:** 2013-05-25

**Authors:** Sana El Mhamdi, Asma Sriha, Ines Bouanene, Arwa Ben Salah, Kamel Ben Salem, Mohamed Soussi Soltani

**Affiliations:** 1 Smoking Cessation Center of Monastir University Hospital, Monastir, Tunisia

## Abstract

**Background:**

Smoking prevalence in adolescents and young adults is substantially elevated in Tunisia. Moreover, there is a lack of knowledge regarding the effectiveness and associated factors in smoking cessation interventions among adolescents and young adults. This study aims at identifying the major factors leading to smoking relapse among adolescents and young adults in the region of Monastir, Tunisia.

**Methods:**

We carried out a prospective cohort study at the smoking cessation center of the University hospital of Monastir, Tunisia. The population study consisted of all adolescents and young adults (15–30 years) consulted during a period of two years (2009 – 2010). A questionnaire was used to explore the patient’s sociodemographic characteristics, smoking history, nicotine dependence (Fagerstrom test) and anxiety / depression (Hospital Anxiety and Depression Scale). A telephone survey was conducted in July 2011 to assess smoking cessation results. A multivariate Cox regression was used to identify predictors of smoking relapses.

**Results:**

A total of 221 adolescents and young adults were included in this study with a mean age of 25.5 ± 3.9 years. At follow up, 59 study participants (26.7%) were abstinent and the overall median abstinence was 2 months. In the multivariate analysis smoking relapse was associated with being an adolescent patient (HR 2.16; 95% CI: 1.54-3.05), medium or higher nicotine dependence at baseline (HR 2.66, 95% CI: 1.06-7.05 and HR 3.12, 95% CI: 1.20-8.12 respectively), not receiving treatment (HR 1.70, 95% CI: 1.25-2.33) and have friend who is a smoker (HR 1.63; 95% CI: 0.96-2.79).

**Conclusion:**

The results of this study provide important information about beneficial effect of smoking cessation support for adolescent and young adults. More efforts must be deployed to deal with contributing factors to smoking relapse.

## Introduction

Smoking is one of the leading public health problems worldwide; it is one of the most preventable causes of death. In Tunisia, tobacco smoking use is substantially elevated. Adult smoking prevalence is estimated at 17.3% according to the “BREATHE study” [1]. This rate remains higher among adolescents rather than adults (from 16 to 29.2%) [2,3]. In addition, cultural factors such as parents who smoke, doctors, nurses, and teachers who smoke in the workplace may add to the creation of a pro-smoking environment [4].

Research suggests that knowledge about the health effects of smoking is a necessary component of tobacco control measures and can result in a postponement of initiation and smoking cessation in youth [5]. Smoking cessation counseling is widely recommended by national and international health organizations, which underscore the great potential for a beneficial intervention at the primary care level. However, smoking cessation outcomes may be affected by smoker characteristics and life context variables [6]. Thus the objective of our study was to identify predictors of smoking relapse among adolescents and young adults in the region of Monastir, Tunisia.

## Methods

### Design

We conducted a prospective cohort study in the city of Monastir in Tunisia from 01/01/2009 to 31/12/2010. The study was carried out in the center of smoking cessation of Monastir University Hospital. All patients aged 15 to 30 years were included in the study, independently of the number of cigarettes smoked per day, number of pack-years or number of previous attempts to quit. All patients were informed and accepted that their data will be used for scientific research. We obtained the approval of the ethical committee of the University Hospital of Monastir (Tunisia).

### Intervention

All smokers received a standardized program for smoking cessation including behavioral counseling and pharmacological treatment. The behavioral counseling consisted of a 45-minute session dealing with aspects of smoking- related morbidity and mortality, key issues of nicotine addiction and the development of a specific action plan for stoppingsmoking. Pharmacotherapy consists of Nicotine Replacement Therapy (NRT), Varenicline (VAR) and bupropion (BUP) and was assigned depending on availability (supplied by the public health system, free of charge) and patient comorbidities and smoking dependence. Pharmacological therapy was prescribed for at least 12 weeks. Patients attended follow-up control visits at weeks 2, 4, 8 and 12 after the scheduled quit day and monthly until the sixth month. Abstinence exceeding sixth months was assessed by telephone survey.

The following two definitions were used in the study:

*Quit date*: starting date of smoking abstinence. This date is chosen by the patient himself

*Point date*: date of the last assessment of smoking status. In this study the point date was set on September 15, 2011.

At baseline a detailed smoking history and health data, was gathered. The questionnaire included items on: Sociodemographic characters (gender, current age, current activity, age of cigarette initiation, age of regular smoking and motivation behind their smoking habit); Daily number of cigarettes and level of smoking addiction using the French version of the Fagerström test for nicotine dependence (FTND) [7] divided into very low dependence (0 – 2), Low / Medium dependence (3 – 6) and high dependence (7 – 10); Anxiety and depressive behavior using the French version of the Hospital Anxiety and Depression Scale (HAD) [8]. A HAD score ≥ 11 corresponds to anxiety or depressive disorders; Friends that are smokers (the investigators decide to consider at least one friend who is smokers in the patient friend’s group; Moderate alcohol consumption (one drink or less per day for women and two drinks or less per day for men during the survey [9]); Number of visits to the medical center and use of pharmacological treatment (corresponding to the use of NRT, VAR and BUP).

At follow up the patient’s smoking status was determined by phone calls focus on the following topics: the current status of smoking, the date of relapse and the causes associated with it. Moreover, self-reported cigarette consumption and possible adverse effects were notified at all follow-up visits.

### Statistical analysis

In the descriptive analysis, categorical variables are expressed as proportions and continuous variables as mean (with standard deviation). Univariate (crude) analysis of variables from the entire population was performed using χ2 test and two-sample test for continuous data. Data were expressed as an odds ratio and 95% confidence interval. All tests used a significant level of 0.05. In this longitudinal study participants could make quit attempts at different times during the follow-up, and thus they also may have different lengths of follow-up to observe relapse. In this case we used a multivariate survival analyses (Cox regression) to identify predictors of relapses. This method allows for differential follow-up and efficiently accommodates missing data due to censoring. Models were fitted, including all factors associated with the outcome, in a stepwise procedure by Kaplan-Meier method. Factors were included in the model when p ≤ 0.25. To be introduced in the multivariate analysis the cutoff point of variables using a visual analogue scale (motivations of smoking behavior) were defined according to ROC curve.

Data was analyzed by SPSS 17.0.

## Results

### Characteristics of the study sample

Among the attendees of the smoking cessation center, 221 adolescents and young adults were tracked during a period of 24 months (from January 2009 to December 2010).

The study sample included 210 men (95%) and 11 women (5%). The patient’s mean age was 25.5 years (SD 3.9). The mean age of smoking initiation was 15.2 years (SD 3.4). A third of our patients (n = 80) consumed alcoholic drinks occasionally (once a month). The mean number of daily cigarette use among our patients was 26 (SD 10.4) with a mean FTND about 6.4 (SD 2). Mean scores for anxiety and depression were 8.4 (SD 4.2) and 5 (SD 3.2) respectively. Basic information and demographic characteristics of the participants were reported in Table 1.

**Table 1:** Sociodemographic characteristics of the study sample at baseline (N = 221)

**Factors**	**Number (%)**	**Means ± SD**
**Age (years)**	–	25.5 ± 3.9
**Gender**		–
Female	11 (5)	
Male	210 (95)	
**Current activity**		–
Profession/training	201 (90.9)	
Unemployed	20 (9.1)	
**Age of smoking initiation**	–	15.2 ± 3.4
**Age of regular smoking**	–	18.5 ± 3.4
**Current alcohol use**		–
Yes	80 (36.2)	
No	141 (63.8)	
**Fagerström score**	–	6.4 ± 2
**Daily number of cigarettes smoked**	–	26 ± 10.4
**Anxiety score**	–	8.4 ± 4.2
**Depression score**	–	5 ± 3.2
**Have friends that are smokers**		–
Yes	64 (29)	
No	157 (71)	
**Number of visits to the medical center for treatment**		–
< 3	70 (31.7)	
3 visits or more	151 (68.3)	
**Use of pharmacological treatment**		–
No	7 (3.2)	
Yes	214 (96.8)	

As depicted in Table 2, several motivations for smoking behavior were assessed using a visual analog scale from 0 to 10. The main motivation for the use of cigarettes was to “deal with stress” with a mean of 7.4(SD 3). Other motivations include automatism with a mean of 7.1 (SD 3.2) and moral support with a mean of 7.3 (SD 3.3).

**Table 2:** Distribution of participants according to the motivation behind their smoking behavior

**Motivation (n)**	**Mean (SD)**	**Minimum**	**Maximum**
Automatic gesture (199)	7.1 (3.2)	2	10
Conviviality (192)	4.8 (3.8)	0	10
Enjoyment (189)	5.4 (3.3)	0	10
Dealing with stress (188)	7.4 (3.0)	1	10
Concentration (201)	6.3 (3.4)	0	10
Moral support (186)	7.3 (3.3)	0	10
Fighting obesity (179)	2.2 (0.7)	0	7

### Predictors of smoking relapse

At follow up, 59 patients (26.7%) were abstinent at the study endpoint with a median abstinence delay of 2 months (95% CI [1.63 – 2.37]). The median delay to smoking relapse was 0.75 months (95% CI [0.42 – 1.07]) for adolescents and 2.50 months (95% CI [2.14 – 2.86]) for young adults (p < 0.01). Table 3 presents the association of each studied factor with the delay of a quit attempt.

**Table 3:** Unadjusted delay in smoking relapse among respondents at follow up (N = 221)

**Factors**	**Median delay (months)**	**95% CI**	**p-value**
**Age**			
Adolescent	0.75	0.42 – 1.07	**< 0.01**
Young adult	2.5	2.14 – 2.86	
**Gender**			
Female	2.0	1.60 – 2.40	**0.52**
Male	1.8	0.70 – 3.30	
**Current activity**			
Permanent profession/training	2.0	1.60 – 2.39	**0.70**
Unemployed	1.9	0.70 – 3.29	
**Age at smoking initiation**			
< 13 years of age	1.2	0.87 – 1.84	**0.21**
≥ 13 years of age	2.0	1.61 – 2.38	
**Age at regular smoking**			
< 18 years of age	1.5	0.88 – 2.11	**0.31**
≥ 18 years of age	2.5	2.10 – 2.89	
**Current alcohol use**			
Yes	2.0	1.07 – 2.92	**0.22**
No	3.0	1.80 – 4.19	
**FTND**			
Very low dependence	18.5	9.30 – 25.20	**0.015**
Low / Medium dependence	3.0	0.85 – 5.14	
High dependence	2.0	1.40 – 2.60	
**Daily cigarette use**			
< 20	2.0	1.71 – 3.82	**0.081**
≥ 20	3.0	1.58 – 2.41	
**Anxiety**			
Yes	2.0	1.01 – 2.98	**0.26**
No	2.5	2.01 – 2.98	
**Depression**			
Yes	1.3	0.54 – 2.06	**0.22**
No	2.0	1.61 – 2.38	
**Smoking friends**			
Yes	1.5	0.80-2.19	**0.011**
No	3.0	2.60-3.40	
**Number of visits to the medical center**			
<3	1.0	0.63-1.36	**<0.001**
3 or more	3.0	1.25-5.66	
**Use of pharmacological treatment**			
No	4.0	2.62-9.13	**<0.01**
Yes	2.0	1.63-2.37	

In the multivariate analysis in Table 4, smoking relapse was associated with being an adolescent patient (HR 2.16; 95% C.I: 1.54-3.05), medium or higher nicotine dependence at baseline (HR 2.66, 95% CI: 1.06-7.05 and HR 3.12, 95% CI: 1.20-8.12 respectively), less than three visits to the medical center, having not received treatment (HR 1.70, 95% CI: 1.25-2.33) and have friend who is a smoker (HR 1.63; 95% CI: 0.96-2.79).

**Table 4:** Factors associated with relapses delay in smoking cessation

***Factors***	**HR**	***95% CI***	***p***
**Fagerström nicotine dependence**			0.040
Very low dependence	1	–	
Low / Medium dependence	2.66	1.06 – 7.05	
High dependence	3.12	1.20 – 8.12	
**Patient’s age**			0.038
Young adult	1	–	
Adolescent	2.16	1.54 – 3.05	
**Number of visits to the medical center**			< 0.001
< 3	1	–	
3 visits or more	0.46	0.33 – 0.64	
**Smoking friends**			0.060
No	1	–	
Yes	1.63	0.96 – 2.79	
**Using treatment**			< 0.001
Yes	1	–	
No	1.70	1.25 – 2.33	

Results of a multivariate Cox regression controlling for gender.

## Discussion

In this study we used data from the Smoking Cessation Center of Monastir University Hospital. Among the 310 monitored smokers, 221 answered the phone survey and the prevalence of abstinence at the endpoint was 26.7%. This kind of center validated secondary prevention strategies to help people quit smoking [10]. This rate is similar to those reported in literature for adolescents and young adults [11,12]. According to literature, younger adults were more likely to be successful quitters than adolescents [13]. In our study we also identified that adolescence may be related to smoking relapses.

A number of studies have indicated that the likelihood of smoking cessation is greater in smokers who had initiated cigarette smoking after the age of 13, in comparison to those who had begun earlier [14]. In our study, the age of smoking initiation as well as the age of regular smoking was not identified as predictor of smoking relapses. The effects of these two ages were perhaps more important in adulthood rather than adolescence among Tunisian youth.

Other findings from this study suggest that smoking cessation might vary also by the level of nicotine dependence. Highly and moderately dependent smokers were found to be less likely to quit than lower dependent ones. Indeed research has indicated that higher nicotine dependence in adolescents and young adulthood predicts later dependence and smoking relapses [15]. Thus, quitting smoking as an adolescent or young adult may substantially alter the risk for later tobacco dependence.

Research has indicated that decreases in the proportion of friends who smoke were robust predictors of cessation [16]. The likelihood of quitting among young people is potentially dependent on the extent of smoking among their peers. Behavioral interventions based on peer education with social networks can help smoking cessation efforts [17].

In our study, having a friend who smoked was not a strongly predictor of smoking relapse. These findings have also been reported by other African countries. For example, Egyptian adolescents were noted to be more influenced by their family’s smoking behavior and perceived adult smoking norms than their peers’ smoking behavior [18].

It is recognized that smoking cessation can be achieved with or without assistance from healthcare but pharmacotherapy may be better in smoking cessation than self-help [19]. NRT and VAR therapy are noted to be effective strategies in smoking cessation especially among adults. Studies proved that they increase the individual’s chance of successfully quitting smoking [20]. Our study, as recent studies [21], proved the effectiveness of smoking cessation therapies in younger smokers. Other studies in developed countries showed that the mean number of cigarettes smoked daily among adolescents and young adults may not exceed 8 or 9 cigarettes per day [22]. Our data showed a higher dependent adolescent population with a mean number of 26 cigarettes smoked daily. With regard to this result a better availability of professional cessation services directed to younger smokers and availability of pharmacological treatment potentially is needed in Tunisia as both treatment and regular follow-up increase long term smoking quitting [23]. A successful treatment may require close follow up, behavioral support and pharmacological therapy [24]. Compared to other developing countries (especially North African countries) current smoking prevalence of Tunisian adolescents is higher (15.3%) and initiation susceptibility reaches 25% among boys thus understanding contributors to smoking initiation and cessation is important for public health interventions [25].

### Limitations and strengths

The important strength of this study is its prospective character. We performed a prospective cohort study over a period of two years to identify predictors of smoking relapse among adolescents and young adults. The advantage of the prospective design is the relative insensitivity for selection and information bias compared with a case–control design. Second, the survival “Cox Regression” allows us to take full account of the censored data. There are also some limitations to our study. The first is the lack of nicotine biomarkers in our context (especially hair and nails analysis) and self-reported smoking cessation. Second, the small number of women number does not allow investigation of interaction and separate analyses for men and women.

## Conclusion

The results of this study confirm the beneficial effect of smoking cessation support for adolescent and young adults. More efforts must be deployed to deal with contributing factors to smoking relapse. Furthermore, health education programmes regarding this potential harm of tobacco use should be implemented for young people.

## Consent

Written informed consent was obtained from the patient for publication of this report and any accompanying files or images.

## Competing interests

The authors declare that they have no competing interests.

## Authors’ contributions

SEM: participate in the redaction of the reaserch protocol / participate in the patient’s follow-up / perform the statistical analysis / write the manuscript. AS: responsable of the patient’s follow-up. IB: participate in the redaction of the reaserch protocol. ABS: participate in the statistical analysis / participate in patient’s follow-up. KBS: responsable of the corrections of the reaserch protocol and statistical analysis. MSS: responsable of the coordination of the reaserch group. All authors read and approved the final manuscript.
